# The rate and assessment of muscle wasting during critical illness: a systematic review and meta-analysis

**DOI:** 10.1186/s13054-022-04253-0

**Published:** 2023-01-03

**Authors:** Brigitta Fazzini, Tobias Märkl, Christos Costas, Manfred Blobner, Stefan J. Schaller, John Prowle, Zudin Puthucheary, Henning Wackerhage

**Affiliations:** 1 Adult Critical Care Unit, The Royal London Hospital, Barts Health NHS Trust, London, UK; 2 Exercise Biology Group, Department of Sports and Health Sciences, Technical University of Munich, Munich, Germany; 3 William Harvey Research Institute, Queen Mary University of London, London, UK; 4 Technical University of Munich, School of Medicine, Department of Anesthesiology and Intensive Care, Munich, Germany; 5 Charité – Universitätsmedizin Berlin, Department of Anesthesiology an Operative Intensive Care Medicine (CVK, CCM), Berlin, Germany; 6 Department of Anesthesiology and Operative Intensive Care Medicine (CVK, CCM), Humboldt-Universität Zu Berlin, Berlin, Germany

**Keywords:** Intensive care unit, Critical illness, Muscle wasting, Muscle atrophy, ICU-acquired weakness, ICU-AW

## Abstract

**Background:**

Patients with critical illness can lose more than 15% of muscle mass in one week, and this can have long-term detrimental effects. However, there is currently no synthesis of the data of intensive care unit (ICU) muscle wasting studies, so the true mean rate of muscle loss across all studies is unknown. The aim of this project was therefore to systematically synthetise data on the rate of muscle loss and to identify the methods used to measure muscle size and to synthetise data on the prevalence of ICU-acquired weakness in critically ill patients.

**Methods:**

We conducted a systematic literature search of MEDLINE, PubMed, AMED, BNI, CINAHL, and EMCARE until January 2022 (International Prospective Register of Systematic Reviews [PROSPERO] registration: CRD420222989540. We included studies with at least 20 adult critically ill patients where the investigators measured a muscle mass-related variable at two time points during the ICU stay. We followed Preferred Reporting Items for Systematic Reviews and Meta-Analyses (PRISMA) guidelines and assessed the study quality using the Newcastle–Ottawa Scale.

**Results:**

Fifty-two studies that included 3251 patients fulfilled the selection criteria. These studies investigated the rate of muscle wasting in 1773 (55%) patients and assessed ICU-acquired muscle weakness in 1478 (45%) patients. The methods used to assess muscle mass were ultrasound in 85% (*n* = 28/33) of the studies and computed tomography in the rest 15% (*n* = 5/33). During the first week of critical illness, patients lost every day −1.75% (95% CI −2.05, −1.45) of their rectus femoris thickness or −2.10% (95% CI −3.17, −1.02) of rectus femoris cross-sectional area. The overall prevalence of ICU-acquired weakness was 48% (95% CI 39%, 56%).

**Conclusion:**

On average, critically ill patients lose nearly 2% of skeletal muscle per day during the first week of ICU admission.

**Supplementary Information:**

The online version contains supplementary material available at 10.1186/s13054-022-04253-0.

## Introduction

Critical illness is defined as the deterioration of an illness resulting in a deranged homeostasis. This leads to life-threatening organ dysfunction requiring advanced organ support techniques, and therefore it is associated with high morbidity and mortality. Both the underlying disease and the causes for the unfavourable course of the disease are diverse and often end in further secondary organ dysfunctions, which are referred to as multi-organ failure. Organ dysfunction, sepsis, prolonged mechanical ventilation, and immobility are risk factors for muscle wasting which leads to ICU-acquired weakness (ICU-AW) [1].

ICU-AW is an umbrella term that describes a bundle of neuromuscular disorders that develop due to admission in the intensive care unit and severe illness [2]. The pathophysiology of ICU-AW is incompletely understood; however, ICU-AW appears to be triggered by critical illness and its severity during the ICU is independent of the underlying primary condition [3–5]. The hallmarks of the ICU-AW are an inflammatory response, bioenergetic dysfunction, altered protein balance, neuronal axon degeneration, changes in muscle histology, and muscle wasting [6, 7]. During critical illness, factors such as immobilisation and altered neuroendocrine responses cause muscle wasting by making protein balance negative [8]. On the other hand, muscle dysfunction is caused by multiple factors including microcirculatory disturbances reducing oxygen supply, bioenergetic mitochondria impairment causing reduced ATP production, and disruptions in the ion channels membrane [9]. These conditions in addition to the patients immobilisation and malnutrition make muscle wasting the dominant phenotype of acquired muscle weakness in critically ill [10].

Patients with critical illness lose muscle mass and muscle function with limited treatment options. Specifically, muscle wasting starts early in the first week of critical illness and patients with multi-organ failure lose more muscle mass than other patients [11]. Observational studies have reported that muscle wasting is associated with a longer stay on ICU [12, 13], and higher ICU [14] and hospital mortality [15]. They also noted that muscle wasting is associated with acquired weakness [16, 17]. However, to date there is no study that has summarised published data on the daily amount of muscle that is lost in ICU patients, the methods used to monitor muscle size in those patients, and on the prevalence of ICU-AW in critically ill patients.

To address this issue, we carried out a systematic review and meta-analysis aiming to answer the following research questions:

1. What is the rate of muscle wasting in critically ill patients?
2. What are the methods used to assess changes in muscle mass in critically ill patients?
3. What is the incidence of ICU-AW in critically ill patients?
4. What are the outcomes (i.e. mortality, mechanical ventilation time, and length of stay) associated with muscle wasting?

## Methods

The study protocol was registered and published on 13 January 2022 on the International Prospective Register of Systematic Reviews (PROSPERO) of the National Institute for Health Research (NIHR) under the ID CRD42022298954. We conducted this systematic review and meta-analysis in accordance with the Joanna-Briggs Institute (JBI) Reviewer’s Manual for Systematic Reviews of Literature [18] and the Preferred Reporting Items for Systematic Reviews and Meta-Analyses (PRISMA 2020) guidelines [19, 20].

### Definitions

The measurement of the rectus femoris is taken by placing the transducer perpendicular to the long axis of the tight on its superior aspect, three-fifth of the distance from the anterior superior iliac spine to the superior patella border. This is the highest point in the tight that the entire rectus femoris cross section can be visualised in a single field. The cross-sectional area is calculated by a planimetric technique after the inner echogenic line of the rectus femoris is outlined by a movable cursor on a frozen image [21].

The quadriceps femoris muscle include vastus medialis, vastus lateralis, vastus intermedius, and rectus femoris.

## Search strategy and selection criteria

### Search strategy

**Fig. 1:**
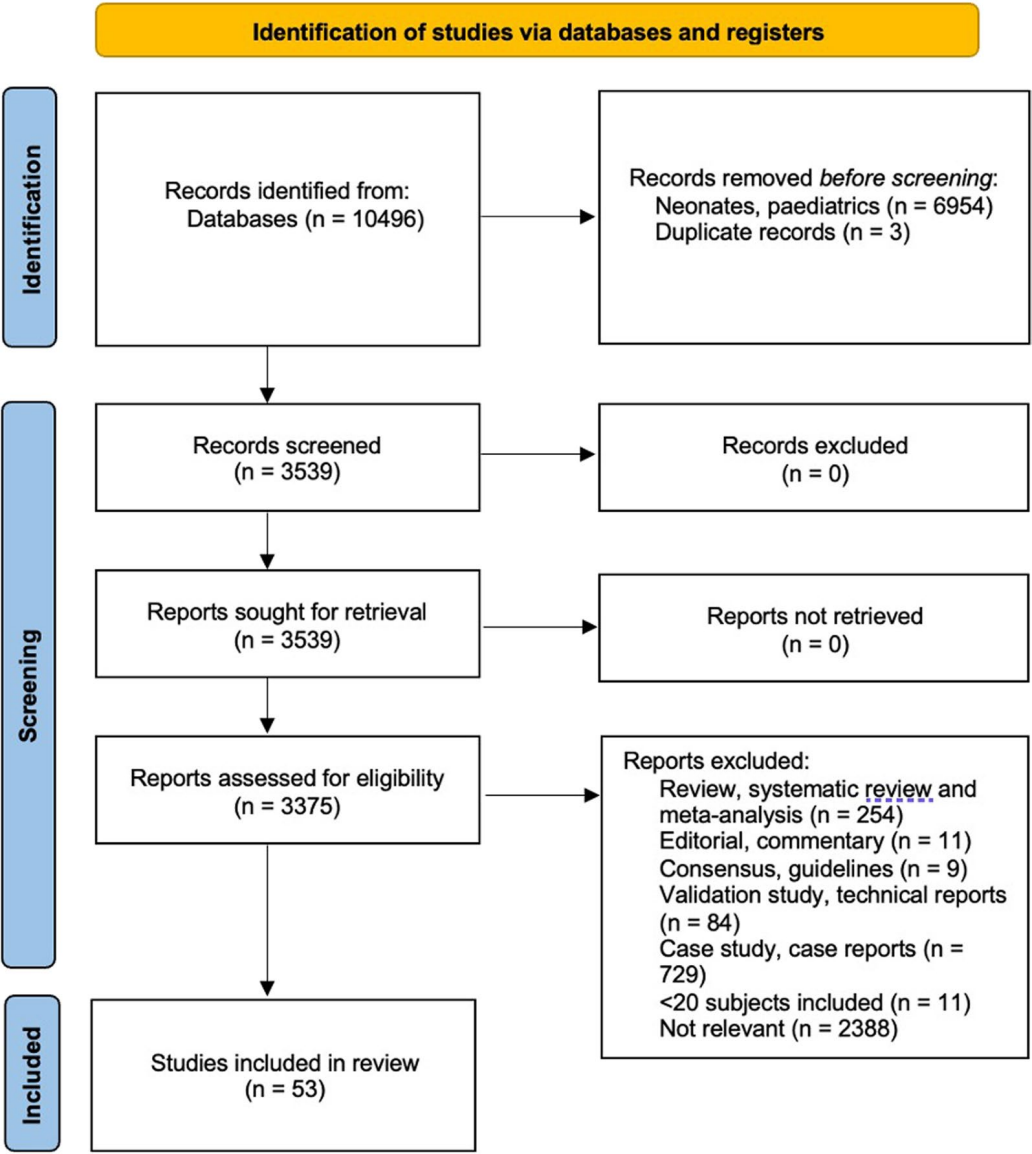
Flow diagram of selected studies according to the PRISMA guidelines

We conducted our search on MEDLINE (National library of medicine: Bethesda, MD) and AMED, BNI, CINAHL, and EMCARE. Studies were also identified and retrieved by citation searching from the references of each relevant study, as reported in Fig. 1.

For the initial literature search, we used a combination of MeSH terms and key terms including (muscle mass OR muscle atrophy OR muscle wast* OR muscle loss OR muscle weakness OR muscle strength OR muscle function OR intensive care unit acquired weakness OR ICU-AW) AND (critical* ill* OR critical care OR intensive care unit OR ICU).

We included studies that were published between 1 January 2000 and 31 January 2022.

The references of all included papers, review articles, commentaries, and editorials on this topic were reviewed to identify other relevant studies which were missed during the primary search. If necessary, we contacted the corresponding authors to obtain data necessary for our study. No language restrictions were applied. Three investigators (BF, TM, and CC) independently screened title and abstracts in duplicate for selection of full-text review. If a decision was not achieved from reading the title and abstract alone, the full text was reviewed. The reviewers also independently reviewed the full text of relevant studies and decided on eligibility. Inter-rater disagreements in the study selection were resolved by consensus or if necessary, by consultation with a senior author (HW). A flow chart of the whole process is presented according to PRISMA guidelines 2020 in Fig. 1.

### Study inclusion and exclusion criteria

Eligible studies included adult women and men (age > 18 years old) admitted to any critical care facility (ICU/high dependency unit [HDU]) receiving invasive or non-invasive mechanical ventilation. Large treatment effects have been reported in studies including fewer patients [22]; hence, we included all peer-reviewed studies enrolling leastwise 20 critically ill patients who had assessment of muscle mass or ICU-AW at any time point after the day of admission or at two time points during ICU stay. We excluded (i) studies published prior 1 January 2000, (ii) reviews and meta-analysis, (iii) book chapters, comments, editorials, (iv) guidelines and consensus report, (v) protocol studies, and (vi) case studies, case reports.

## Data extraction

Three reviewers (BF, TM, and CC) extracted the following information from each publication into an Excel file: date of publication, country, study design, number of included patients, age, gender, clinical features, laboratory findings, severity and outcome of the disease (including health-related quality of life, cognitive status, mental health, physical function, muscle and/or nerve function, and pulmonary function). Data extraction was performed in duplicate by three authors acting independently (BF, TM, and CC). A flow chart of the whole process is presented according to PRISMA guidelines 2021 in Fig. 1. The characteristics of the studies included are presented in Table 1.

**Table 1:** Characteristics of included studies assessing muscle wasting

Author/References	Design/Country/Setting	No. of Population	Inclusion criteria	Tool	Body site	Timing	Muscle mass loss	Outcomes
1. Lambell [45]	RC single centre Australia ICU	32 Trauma, medical, surgical	> 18 yo, who had CT scans before admission with routine care and a second or multiple CT scan ≥ 7 days later. Patients included if both CT scans were appropriate for analysis of SMA and if the predominant nutrition route was enteral and/or parenteral (planned > 70% requirements), due to oral intake not being routinely recorded in a quantifiable manner	CT	Skeletal muscle CSA at L3 level	CT scan at week 1 (day 0–7) and second CT scan ≥ 7 days later	SMA loss in 7 days MD: −21.9 [−29.9 to −13.9] cm^2^, (149.9 ± 38.8 vs. 127.9 ± 38.4 cm^2^), *p* < 0.001 %SMA change per day: −1.27 ± 0.88% cm^2^	Not reported
2. Lee [15, 69]	PC Single Centre Malaysia ICU	86 Cardiovascular, respiratory, gastrointestinal, neurology, sepsis, trauma, metabolic, renal, immunocompromised	Consecutive patients > 17 years old, expected to stay > 96 h on ICU. Patients with ‘normal’ baseline muscle status	USS	Quadriceps muscle layer thickness (QMLT), RF CSA, VI, pennation angle (PA) and fascicle length (FL)	Day 1 (within first 48 h), 7, 14, 22 of ICU admission	%QMLT compared to baseline: at day 7: −8.61 ± 19.44 at day 14: −15.63 ± 23.75 %RF CSA compared to baseline: at day 7: −9.81 ± 19 at day 14: −22.73 ± 20	Every 1% loss of QMLT over the first week of critical illness was associated with 5% increase in 60-day mortality (Adjusted odds ratio [AdjOR] 0.950 for every 1% less QMLT loss, 95% CI 0.90,0.99; *p* = 0.023
3. Toledo [14]	PC single centre Brazil ICU	74 Sepsis, stroke, lung transplant, cardiac insufficiency	Patients > 18 years old, needing mechanical ventilation for ≥ 48 h	USS	Quadriceps muscle thickness	Day 1, 3, 7	% Quadriceps muscle thickness decrease from day 1 to day 7 Right leg: 15% (± 19.5%) Left leg: 12.7% (± 16%)	Predictor of survival: cut-off value in muscle thickness of ≤ 1.64 cm on day 7 (HR = 0.7, 95% CI, 0.582–0.801, sensitivity 81%, specificity 63%). Higher probability to remain on mechanical ventilation in patients with 1.64 cm loss of thigh muscle thickness on day 7, HR: 2.1 (95% CI, 1.1–3.8) higher than their counterparts (*P* = 0.017). Greater loss of thigh muscle thickness on day 7 for worst ICU survival (HR: 3.7; 95% CI, 1.2–11.5) and hospital survival (HR: 4.5; 95% CI, 1.5–13.7)
4. Zhang [26]	PC Single centre China ICU	37 Sepsis, pneumonia, severe pancreatitis, liver failure, renal failure, cardiac dysfunction, surgical	Patients aged ≥ 18 years with an anticipated ICU stay of at least 2 days	USS	RF thickness and CSA, VI and BB muscles	Day 1, 4, 7, 10	% not reported	Not reported
5. Borges [27]	PC Single centre Brazil ICU	45 Severe and septic shock	Patients > 18 yo with sepsis or severe septic shock within 24 h of admission	USS	RF CSA	Day 2, 4, 6, at ICU discharge and hospital discharge	RF CSA: day 2: 5.11 ± 0.85cm^2^ versus day 6: 4.49 ± 0.84cm^2^; *P* = 0.001 Daily RF CSA loss: 1.2% During ICU stay average muscle loss of 13.5% compared to baseline	RF CSA in patients who underwent mechanical ventilation versus those without mechanical ventilation, *P* = 0.080. RF CSA during hospital stay in mechanically ventilated 17.25% versus patients without ventilation 10.76%, P = 0.001
6. Dimopoulos [13]	PC Single centre Greece ICU	165 Cardiac surgery	Patients > 18 yo admitted to cardiac ICU within 24 h of cardiac surgery	USS	RF thickness	Day 1, 3, 5, 7	RF mass (cm): D1: 1.37 ± 0.25 D3: 1.2 ± 0.5 D5: 1.25 ± 0.52 RF + VI mass(cm): D1: 2.58 ± 0.34 D3: 2.41 ± 0.94 D5: 2.37 ± 0.8 In 5 days RF mass loss by 2.2% [(95%CI: −0.21 to 0.15), P = 0.729] and RF + VI mass loss by 3.5% [(95% CI: −0.4 to 0.22), *P* = 0.530]	RF + VI mass < 2.5 cm on D1: longer ICU length of stay (47 ± 74 h vs 28 ± 46 h, *P* = 0.02) and ventilator time (17 ± 9 h vs 14 ± 9 h, *P* = 0.05). ICU-AW versus no ICU-AW on D3: longer ventilation (44 ± 14 h vs 19 ± 9 h, *P* = 0.006) and ECMO (159 ± 91 min vs 112 ± 71 min, *P* = 0.025)
7. Kemp [12]	PC Single centre UK ICU	20 Cardiac surgery	Adults > 18 yo with elective aortic operation requiring admission to the ICU as identified by the surgical team	USS	RF CSA	Day before surgery and day 1, 3, 7 after surgery	RF CSA (cm^2^) at D0: 6.85 ± 1.45 (5.4–8.3) D7: 6.3 ± 1.45 (4.85–7.75) RF CSA mass loss: 8% (6.6–10.2)	Muscle loss > 10% was associated with longer ICU length of stay (*P* = 0.038), hospital length of stay (*P* = 0.014), mechanical ventilation time (*P* = 0.05)
8. Mayer [16]	PC Single centre USA ICU	41 Sepsis or acute respiratory failure	Adults > 18 yo, diagnosis of acute respiratory failure or sepsis of any origin anticipated to survive and spend > 3 days on ICU, enrolled within 48 h of admission	USS	RF and TA CSA, muscle thickness (mT), echo intensity (EI)	Day 1, 3, 5, 7	RF mT: D1 0.98 ± 0.3 versus D7 0.81 ± 0.27, *P* = 0.0316 RF CSA: D1 2.99 ± 0.99 versus D7 2.47 ± 0.88; *P* = 0.0253 RF EI: D1 91 ± 24.9 versus D7 99.1 ± 27.6; *P* = 0.081 TA mT: D1 2.01 ± 0.36 versus D71.82 ± 0.31, *P* < 0.001 TA CSA: D1 5.3 ± 0.89 versus D7 4.71 ± 0.95, *P* < 0.001 TA EI: D1 82.7 ± 21.2 versus D7 96.7 ± 22.6; *P* = 0.002 Changes from D1 to 7 RF mT: 20.1% (12–26) RF CSA: 18.5% (11–23) RF EI: 10.5% (5–20) TA mT: 9.1% (5–12) TA CSA: 8.1% (5–15) TA EI: 15.4% (7–28)	RF EI in first 7 days of ICU admission predictor of ICU-AW (area under curve = 0.912)
9. McNelly [44]	RCT Multi-centre UK ICU	121 mechanically ventilated patients	Adult (> 18 years), expected to be intubated and ventilated for ≥ 48 h; requiring enteral nutrition via nasogastric tube; multi-organ failure (Sequential Organ Failure Assessment [SOFA] score > 2 in ≥ 2 domains at admission); likely ICU stay ≥ 7 days and likely survival ≥ 10 days	USS	RF CSA	At Day 1, 7, 10 in both groups	Intermittent feed Day 7 −12.9%(95%CI −17.1 to –8.7) Day 10 −18.7% (95% CI −29.8 to −7.6) Continuous feed Day 7: −14.7% (95% CI −19.5 to 9.9) Day 10: −20.6% (95% CI −31.0 to 10.2) *P* value *P* = 0.431 *P* = 0.337	Safety profiles, gastric intolerance, physical function milestones, and discharge destinations did not differ between groups
10. Nakamura [46]	RCT Single centre Japan ICU	117 Medical and surgical	Patients admitted to ICU	CT	Femoral muscle volume	Day 1 and 10	Femoral muscle volume loss was 12.9 ± 8.5% in the high-protein group and 16.9 ± 7.0% in the medium-protein group, with significant difference (p = 0.0059)	For critical care, high-protein delivery provided better muscle volume maintenance, but only with active early rehabilitation
11. Nakanishi [28]	PC Multi-centre Japan ICU	56 Respiratory failure, heart failure, sepsis, cardiac arrest, trauma, neurologic	Consecutive adult > 18 yo, expected to remain in ICU > 5 days. Patients were prospectively recruited within 12 h of ICU admission	USS	RF CSA	Day 1, 3, 5, 7	RF CSA loss: –8.6 ± 4.9% on D3, –13.8 ± 5.9% on D5, –18.2 ± 5.6% on D7, respectively (*p* < 0.01)	Not reported
12. Nakanishi [29]	PC Multi-centre Japan ICU	64 Respiratory failure, sepsis, post-cardiac surgery, heart failure, cardiac arrest, trauma, neurologic	Expected mechanical ventilation > 48 h, stay in ICU > 5 days	USS	BB CSA, RF CSA	Day 1, 3, 5, 7 and ICU discharge	BB CSA decreased by 6.0% (95% CI, 4.4–7.6%) D3, 11.0% (95% CI, 9.3–12.7%) D5, and 15.6% (95% CI, 13.5– 17.6%) D7 (p < 0.01) RF CSA decreased by 6.2% (95% CI, 3.3%–9.1%) D3, 12.9% (95% CI, 9.8–15.9%) D5, and 17.1% (95% CI, 13.4%– 20.7%) D7; (*p* < 0.01) BB CSA loss: 2.24% per day, BB CSA loss: 15.6% per week, RF CSA loss: 2.44% per day, RF CSA: 17.1% per week	BB and RF muscle atrophy did not predict in-hospital mortality on day 3 (*P* = 0.70 and *P* = 0.53, respectively). BB muscle loss predicted mortality on days 5 (*P* = 0.02) and 7 (*P* = 0.01). RF muscle atrophy on days 5 and 7 predicted mortality (*P* = 0.02 and *P* = 0.01, respectively)
13. Borges [30]	PC Single centre Brazil ICU	37 Severe sepsis or septic shock	Patients > 18 yo diagnosed with severe sepsis or septic shock within 24 h of evolution	USS	RF CSA	Day 2, 4, 6, ICU discharge and hospital discharge	RF CSA loss: −5.20 ± 0.47 on D2, −4.4 ± 0.45 on ICU discharge and 4.36 ± 0.42 on hospital discharge, (*P* < 0.05) RF CSA: −1.45% per day; −14.5% ± 7.6 in 10 days	No difference in RF CSA between patients who underwent mechanical ventilation and in those without; *P* = 0.08
14. Dusseaux [47]	RC Single centre France ICU	25 Sepsis, septic shock, acute pancreatitis, cardiac arrest, pneumonia, endocarditis	> 18yo, in ICU for at least 7 days, required mechanical ventilation during their ICU stay, and had abdominal CT scans within the first 48 h of admission to ICU (CT 1: initial assessment) and 7 to 14 days after (CT 2: late assessment CT 2)	CT	Skeletal muscle radiodensity, skeletal muscle mass CSA at L3 vertebra	CT 1: within the first 48 h of admission CT 2: 7 to 14 days later	SMM (cm^2^/m^2^): CT 1 48.73 ± 12.57; CT 2 46.64 ± 10.64 SMD: CT 1 34.86 ± 10.46, CT 2 33.56 ± 7.67 SMM loss: −2.09 (± 6.96); p = 0.183 over 7–14 days SMD loss: −1.3 ± 8.53 over 7–14 days	No significant correlation was observed between mortality outcome and SMM [*P* = 0.289; OR 95% CI: 0.93 (0.81–1.060)] or SMD [*P* = 0.091; OR 95% CI: 1.12 (0.98–1.28)]
15. Haines [48]	RC Single centre UK ICU	10 7 Trauma	All trauma admissions admitted to the adult ICU either directly or via the operating theatre	Urea/creatinine ratio CT	Total abdominal muscle CSA measured at the level of the third lumbar (L3) vertebrae, and psoas muscle CSA was calculated at the L4 level	CT 1: on admission CT 2: within 1–9 days or after 10 days of ICU stay	At the second CT urea/creatinine ratio negatively correlated with L4 psoas and L3 muscle cross-sectional areas (R2 0.39, *p* < 0.001)	
16. Nakanishi [31]	PC Single centre Japan ICU	21 surgical, non-surgical	Adults > 18yo expected mechanical ventilation > 48 h, stay in ICU > 5 days	USS, BIA	Combined BB and RF CSA	Day 1, 3, 5, 7, 10	Muscle mass: on D3 −9.2% (95% CI, 5.9–12.5%), on D5 −12.7% (95% CI, 9.3–16.1%), on D7 −18.2% (95% CI, 14.7–21.6%), on D10 −21.8% (95% CI, 17.9–25.7%) (*P* < 0.01) Muscle loss: −2.6% per day; −18.2% per week	Not reported
17. Trung [32]	PC Single centre Vietnam ICU	79 Tetanus	Patients ≥ 16 y of age with a clinical diagnosis of generalized tetanus and within 48 h of ICU admission	USS	RF CSA	Day 1, 7, 14, at hospital discharge	RF CSA loss: −7.43 ± 3.17 at D7, −11.59 ± 4.52 at D14, −13.2 ± 5.4 at discharge Muscle loss between admission and discharge *P* < 0.01	Not reported
18. Wandrag [33]	PC Multi-centre UK ICU	43 Pneumonia, cardiology/cardiac surgery, neurology/neurosurgery, sepsis, septic shock, major trauma, traumatic brain injury, gastroenterology, gastrointestinal surgery, HIV, multi-organ failure, renal failure	Patients > 18yo, anticipated to be ventilated > 48 h	USS	Combined muscle depth BB, forearm (flexor compartment of muscle) and thigh (rectus femoris and vastus intermedius)	Day 1, 3, 7 and 14	Total muscle depth (cm): D1: 7.6 ± 3.7, D7: 6.5 ± 3.1; MD (cm): −1.1 (1.5–0.7), *P* < 0.0001	
19. Hadda [34]	PC Single centre India ICU	70 Sepsis	Adults > 18 years old, diagnosis of sepsis (non-surgical)	USS	BB thickness and quadriceps muscles	Day 1, 3, 5, 7, 10, 14 and then weekly until discharge or death	On day 7 percentage muscle thickness loss [median (IQR)] BB: 7.61 (− 1.51, 32.05) %; *P* < 0.001 Quadriceps: 10.62 (− 1.48, 32.06) %, *p* < 0.001); *P* < 0.001	Decline in muscle thickness was significantly higher among patients with worse outcome at 90 days
20. Hayes [35]	PC Single centre Australia ICU	25 ARDS, bridge to transplant, pulmonary hypertension, cardiac failure/infarction, cardiac arrest	Patients > 18 yo expected to be on ECMO > 24 h or > 5 days in ICU prior to recruitment	USS	RF CSA	At baseline, day 10, day 20	RF CSA loss: 4.2 ± 1.3 at D1, 3.4 ± 1.1 at D10 compared to baseline: RF CSA −19.2% [95% CI, − 13.7 to − 24.8%], *P* < 0.001 at day 10; −30.5% [95% CI, − 24.1 to − 36.9], *P* < 0.001 at day 20	Not reported
21. Katari [36]	PC Single centre India ICU	100 Mixed medical and surgical	Patients 18–90 years old, anticipated ICU stay > 7 days	USS	Total anterior thigh thickness, RF thickness, and combined thickness of VI and RF	Day 1, 3, 7	RF thickness: D1: 1.37 ± 0.41, (0.96) D3: 1.26 ± 0.41, D7: 1.22 ± 0.47; *P* < 0.001 respectively RF thickness: −11 (± 38.5)% at D7 compared to baseline	Not reported
22. Nakanishi [37]	PC Single centre Japan ICU	28 Mixed ICU patients	Expected mechanical ventilation > 48 h, stay in ICU > 5 days	USS	BB and RF thickness and CSA	Day 1, 3, 5, 7	Loss compared to baseline: BB thickness at D7 −13.2%; *P* < 0.01 BB CSA at D7-16.9%; *P* < 0.01 RF thickness at D7: −18.8% RF CSA at D7: −20.7%	Not reported
23. Palakshappa [17]	PC Single centre USA ICU	29 Medical patients with sepsis and shock or respiratory failure	Admitted to the medical ICU with a diagnosis of sepsis complicated by respiratory failure or shock requiring vasopressors for a minimum of 6 h, and an anticipated ICU length of stay > 48 h	USS	RF CSA Quadricep muscle thickness	On Day 0 and Day 7	RF CSA decreased by 23.2% Quadriceps thickness decreased by 17.9%	Quadriceps muscle thickness shows a weak correlation with the strength RF CSA depicts a moderate correlation with the strength
24. Pardo [24]	PC Single centre France ICU	29 Mixed ICU patients	> 18 years old, expected ICU stay > 7 days, patients to receive muscle US as part of usual care	USS	Quadriceps femori muscle thickness	Day 1, 3, 5, 7, 21	Quadriceps femori at admission: 1.72 [95% CI, 1.62; 2.13], D7: 1.45 [95% CI, 1.24; 1.665] *P* < 0.01, D21: 1.30 [95% CI, 0.80; 1.48] *P* < 0.01 Quadriceps femori loss: 16% over a week	Not reported
25. Silva [38]	PC Single centre Brazil ICU	22 TBI	Patients 18–60 yo and mechanically ventilated	USS	TA, BB and RF muscle thickness	Day 1, 7, 14	Muscle wasting at D14 compared to baseline: RF: −22% *P* = 0.0001, TA: −19% *P* = 0.0001, BB: −12% *P* = 0.0004	Not reported
26. Annetta [39]	PC Single centre Italy ICU	38 Trauma	Trauma patients with an injury severity score (ISS) exceeding 25, admitted to ICU within few hours after the injury. Only well-nourished, previously healthy subjects, aged 18–59 yo	USS	RF and TA CSA	Admission day, 5, 10, 15, 20	RF CSA (cm^2^): D0: 6.1 [5.1–7.3], D5: 5.9 [4.8–6.3], D10: 5.1 [4.3–6.2], D15: 4.6 [3.8–5.3], D20: 3.5 [3.2–4.7] AT CSA (cm^2^): D0: 5.6 [4.5–6.4], D5: 4.8 [3.7–5.6], D10: 4.0 [3.7–5.2], D15: 4.0 [3.3–4.8], D20: 4.2 [3.4–4.7] Overall 45% reduction in RF CSA during the first 20 days of ICU stay; 15% loss from day 5 to 10, 12% from day 10 to 15, 21% from day 15 to 20 TA CSA 22% loss during the overall ICU stay, *P* = 0.30	Not reported
27. Puthucheary [40]	PC Multi-centre UK ICU	43 Surgical and medical	All patients were recruited within 24 h of admission to a university hospital and a community hospital and were expected to survive intensive care unit (ICU) admission after being invasively ventilated for over 48 h and in the ICU longer than 7 days	USS	RF thickness and RF CSA	Day 1, 7, 10	RF thickness Day 7: −5.88 (−11.69, −0.06) Day 10: −9.65 (−15.43, −3.84 (*P* = 0.031) RF CSA Day 7: −13 (−16.5, −9.48) Day 10: −17.72 (−21.15; −14.29) (*P* = 0.004	ΔRF_CSA_ was greater in those with knee extensor weakness than those without (20.7% [95% CI, 13.7–27.7] vs. 8.4% [95% CI, 2.5–14.3], respectively; *P* = 0.012). ΔThickness did not differ between these groups (12.6% [95% CI, 0.94–24.2] vs. 12.1 [95% CI, 2.7–21.5], respectively; *P* = 0.95). In a bivariable logistical regression, ΔRF_CSA_ was associated with knee extensor weakness (odds ratio, 1.101 [95% CI, 1.011–1.199]; *P* = 0.027), but Δthickness was not (odds ratio, 1.001 [95% CI, 0.960–1.044]; *P* = 0.947)
28. Segaran [41]	PC Single centre UK ICU	39 Surgical, medical, trauma	Patients > 18 yo, BMI > 19 kgm^−2^, expected to be mechanically ventilated > 48 h, and artificially fed	USS	Muscle depth of BB, forearm and thigh	Day 1, 3, 5, 7, 12, 14	Muscle loss per day:2.93%, at D7: 20.53%	Not reported
29. Turton [42]	PC Single centre UK ICU	22 Mechanically ventilated critically ill patients	Patients who > 18 years of age who were assented within 24 h of being intubated and admitted to the participating intensive care units were included in the study	USS	*Pennation Angle* and *Fascicle Length* and *Muscle thickness* Upper Limb: Right Elbow Flexor Compartment Lower Limb: Right Vastus Lateralis The right medial head of the gastrocnemius	On days 1, 5 and 10	Elbow flexor compartment and gastrocnemius muscle thickness did not significantly change Vastus Lateralis pennation angle and muscle thickness significantly reduced by day 5 Fascicle length did not significantly change for all three muscle groups	Muscle thickness and architecture of vastus lateralis undergo rapid changes during the early phase of admission to a critical care environment
30. Parry [25]	PC Single centre Australia ICU	22 Mixed medical and surgical	Adults ventilated > 48 h, remain at least 4 days in ICU	USS	RF thickness, vastus lateralis thickness, VI thickness, RF CSA	Baseline (day 1), day 3, day 5, day 7, day 10	Compared to baseline: RF Thickness: D3: −8.7%, D5: −16.6%, D7: −24.9%, D10: −30.4%.; VI Thickness D3: −1.3%, D5: −18.1%, D7: −20.0%, D10: −29.7% VL thickness D3: −0.2%, D5: −5.7%, D7: −6.0%, D10: −14.1% RF CSA D3: −1.0%, D5: −11.8%, D7: −16.8%, D10: −29.9%	Correlation between ICU discharge and RF, VI, VL thickness (*P* < 0.05)
31. Jung [49]	RC Single centre France ICU	23 Mixed ICU patients	Admitted to ICU and had CT scan before admission, CT scan during ICU, at least one measure of diaphragmatic contractility	CT scan	Psoas volume, CSA of skeletal muscles at L3 vertebra examination with 64-section spiral CT	Baseline and 25 days after ICU admission	Psoas volume baseline:272 ± 116, D25: 233 ± 108; *P* < 0.01 Skeletal muscle CSA cm2/m2 baseline: 17.1 ± 5.4, D25: 16.1 ± 5.2 Psoas loss: 14.34% skeletal muscle CSA loss: 5.85%	Not reported
32. Puthucheary [11]	PC Single centre UK ICU	63 Sepsis, trauma, intracranial bleeding, acute liver failure, cardiogenic shock	Patients > 18 yo, anticipated to be intubated > 48 h, spend > 7 days in critical care, and to survive ICU stay	USS; 28 patients were assessed by USS, ration protein DNA, histopathological analysis	RF CSA, biopsy, histological samples	Day 1, 3, 7, 10	RF CSA mm2 at D1: 514 (464–566), D3: 495 (442–549), D7: 450 (402–498), D10: 423 (378–469) From days 1 to 7 (− 12.5% [95% CI, − 15.8% to − 9.1%]; *P* = 0.002), and to day 10 (− 17.7% [95% CI, − 20.9% to − 4.8%]; *P* < 0.001) In 28 patients assessed by all 3 methods on days 1 and 7, the rectus femoris cross-sectional area decreased by 10.3% (95% CI, 6.1% to 14.5%), the fibre cross-sectional area by 17.5% (95 CI%, 5.8% to 29.3%), and the ratio of protein to DNA by 29.5% (95% CI, 13.4% to 45.6%)	Not reported
33. Reid [43]	PC Single centre UK ICU	50 Sepsis, cardiac, respiratory failure, multiple trauma, head injury, head injury, medical	Patients > 18yo admitted to the ICU for ventilatory support > for 5 days or longer	USS	Mid-upper arm circumference and muscle thickness	1–3-day intervals between 5 and 39 days (median 7 days)	Muscle thickness at baseline: 4.5(2.6–6.8); change at D7: −0.57(0.2–2.3) Muscle loss: 1.6%(0.2–5.7) per day, 12.05%(0–46.7)	Not reported

*PC* prospective cohort, *RC* retrospective cohort, *RCT* randomised controlled trial, *ICU* intensive care unit, *CT* computed tomography, *USS* ultrasound sonography, *CSA* cross-sectional area, *SMA* skeletal muscle area, *MD* median difference, *RF* rectus femoris, *VI* vastus intermedius, *BB* bicep brachii, *TA* tibia anterior, *mT* muscle thickness, *D* day, *ICU-AW* intensive care unit acquired weakness

### Risk of bias assessment

Three reviewers (BF, TM, and CC) independently assessed the risk of bias using the Newcastle–Ottawa Scale (NOS) for observational studies [14], and the Cochrane Risk of Bias tool (ROB2) was used for assessing randomised controlled trial [23]. Risk of bias across studies was assessed using the approach outlined by the Grading of Recommendations Assessment Development and Evaluation (GRADE) working group [15, 16]. Any disagreements were recorded and resolved by involvement of an additional reviewer.

## Data synthesis and analysis

A narrative and tabular synthesis of the findings from the included studies was provided. Data were grouped into the main outcomes above specified. Numerical data on the long-term outcomes above specified were collected for quantitative analysis.

### Statistical analysis

Mean and standard deviation (SD) or median and interquartile range (1st quartile to 3rd quartile) were used for numerical data if appropriate, while odds ratio (OR) with 95% confidence interval (CI) was used for categorical data. For data presenting median and interquartile range (IQR) or median and range, mean and standard deviation (SD) were transformed according to standard equations [17–19]. The studies included for meta-analysis were pooled together using the random-effects model accounting for the incidence. The results were presented in forest plots. Heterogeneity among studies was evaluated using the Tau^2^ test, *I*^2^ statistics, and Cochrane Q. A *p* value < 0.05 was considered as evidence of publication bias. Analysis of data was performed using the statistical software packages Review Manager 5.4 (RevMan 5.4.1®) and OpenMeta [Analyst]®.

## Results

We identified 10,496 studies through our literature search. After removing duplicates and publications that did not fit our inclusion criteria, we were left with 53 publications. Of these, 33 quantify muscle wasting over time with 4 studies measuring muscle wasting and ICU-AW and 20 studies assess ICU-AW only. See Fig. 1 to appreciate the flow diagram of the studies included.

Overall, the publications reported data on 3251 patients, 1773 (55%) on muscle wasting, and 1478 (45%) for ICU-acquired weakness. We found 1 randomised controlled trial and 43 single-centre and 8 multi-centre observational studies across Australia, Asia, USA, South America, and Europe. Studies’ characteristics are summarised in Tables 1 and 2.

**Table 2:** Characteristics of included studies assessing ICU-acquired weakness

Author/References	Design/Country/Setting	No. of Population	Inclusion criteria	Exclusion criteria	Tool	Score	Prevalence of ICU-AW
1. Van Aerde et al. [50]	RC Single centre Belgium ICU	50 COVID-19	Adult patients (> 18 years old) requiring mechanical ventilation	N/A	Clinical examination	MRC sum score	(36/50) 72%
2. Ballve et al. [51]	PC Single centre Brasil	111	Adult patients (> 18 years old), mechanically ventilated for ≥ 24 h	Pre-existing neurological conditions (central or peripheral nervous system disease, stroke), orthopaedic or traumatic limitations	Clinical examination	MRC sum score	(66/111) 59%
3. Nguyen et al. [52]	PC Single centre Vietnam	133	> 15 years old, residents of ICU for at least 10 days	N/A	Clinical examination	MRC sum score and neuropathy limitation scale (ONLS)	(73/133) 55%
4. Parry et al. [25]	PC Single centre Australia	60	Mechanical ventilation for at least 48 h	Pre-existing neurological conditions (central or peripheral nervous system disease, stroke)	Clinical examination	MRC sum score and a new 4-point scoring system as well as handgrip dynamometry Diagnosis: MRC-SS: < 48/60, MRC 4-point score: < 24/36	(25/60) 42%
5. Hough et al. [53]	PC Single centre USA	30	> 3 days of mechanical ventilation	Pre-existing neurological conditions (central or peripheral nervous system disease, stroke), language barriers	Clinical examination	MRC sum score Diagnosis: MRC < 48/60	(6/30) 20%
6. Brunello et al. [54]	PC Single centre Switzerland	39	Systemic Inflammatory Response Syndrome (SIRS) diagnosis, Mechanical Ventilation for > 2 days	Pre-existing neurological conditions, paediatric patients	Clinical examination	Physical and Neurological examination: assessment of 10 muscle groups, skin sensorimotor response and tendon reflexes Diagnosis: Modified MRC Score of < 35/50	(13/39) 33%
7. Carstens et al. [55]	PC Single centre Germany	56	Patients on mechanical ventilation with a SAPS II score of ≥ 20	< 18 years old, patients diagnosed with other known myopathies or neuropathies, thrombocytopenia	Electrophysiological examination	Diagnosis: CMAP < 3 mV in at least one investigation before awakening	(34/56) 61%
8. Sharshar et al. [57]	PC Multi-centre France	115	Mechanical ventilation for > 7 days	Pre-existing neuromuscular conditions, or other myopathies	Clinical examination	MRC sum score Diagnosis: MRC < 48/60	(75/115) 65%
9. Nanas et al. [58]	PC Single centre Greece	185	Mechanical ventilation for at least 10 days	Muscle weakness before ICU admission, muscle relaxant administration, pre-existing neuromuscular conditions	Clinical examination	MRC sum score Diagnosis: MRC < 48/60	(44/185) 23.8%
10. Ali et al. [59]	PC Multi-centre USA	136	Age ≥ 18 years old, mechanical ventilation for ≥ 5 days	Mechanically ventilated before referral to ICU, limb amputation ≥ 2 parts, subject unable to communicate	Clinical examination	MRC-ss and handgrip dynamometry Diagnosis: MRC < 48/60	(35/136) 25.5%
11. Latronico et al. [60]	PC Multi-centre Italy	92	> 15 years old, score 35–70 in SAPS II	Pre-existing neuromuscular conditions, multiple organ failure, amputations, fractures, oedema in legs	Electrophysiological examination	CMAP or SNAP amplitude reduced by > 2 Standard Deviations (SD) of normal limits	(28/92) 30.4%
12. Villar et al. [61]	PC Single centre Spain	30	Mechanical ventilation for >or = 48 h, IV corticosteroids (> or = 240 mg methylprednisolone) during admission, admitted as a result of COPD exacerbation	> 80 years old, comorbidities of cardiogenic, renal or pulmonary origin	Electrophysiological examination	Electromyography after weaning from ventilation Muscle biopsy obtained if the patients diagnosed with myopathy from the electrophysiological examination	(9/30) 34.6%
13. Bednarik et al. [62]	PC Single centre Czech Republic	51	SOFA score grades 3 or 4 in two organ systems, admission in ICU within 24 h of critical illness	Pre-existing neuromuscular conditions	Clinical examination and electrophysiological examination	Clinical examination: daily from the day of admission until day 28 Performance of electrophysiological analysis twice: the first week of admission and the fifth week Diagnosis: MRC grade ≤ 2 in examined muscles CIPM diagnosis if there are fibrillation potentials, reduced CMAP amplitude	Clinical examination: (17/51) 27.9% Electrophysiological examination: (35/51) 57.4%
14. Montero et al. [64]	PC Single centre Spain	26	Patients diagnosed with septic shock, mechanically ventilated for at least one week	Between 18 and 80 years old, pre-existing neuropathies or myopathies, infected with HIV, renal failure	Electrophysiological examination once the patient weans from mechanical ventilation	reduction in CMAP and SNAP amplitudes	(34/64) 53.1%
15. Bercker et al. [65]	RC Single centre Germany	45	Patient diagnosed with ARDS	Pre-existing neuromuscular conditions	Clinical examination and electrophysiological examination	Clinical assessment using MRC-SS Electrophysiological examination at early days of admission	(27/45) 60%
16. Jonghe et al. [66]	PC Multi-centre France	95	Mechanical ventilation for ≥ 1 week	Pre-existing neuromuscular conditions, language barrier	Clinical examination and electrophysiological examination	Clinical assessment using MRC-SS once patient awake Electrophysiological examination at day 10 Diagnosis: MRC-SS < 48, Reduced CMAP	(24/95) 25.3%
17. Letter et al. [3]	PC Single centre Netherlands	98	Mechanical ventilation for at least 4 days	Pre-existing spinal cord injuries or pre-existing diagnosed myopathy	Clinical examination and electrophysiological examination	Clinical examination twice weekly during admission Electrophysiological nerve conduction studies on days 4, 11, 25 after initiation of mechanical ventilation Diagnosis: Motor sum score < 26 with absent tendon reflexes, CMAP < 2.6 mV (peroneal nerve) and CMAP < 4.2 mV (ulnar nerve)	(32/98) 33%
18. Druschky et al. [67]	PC Single centre Germany	28	Mechanical ventilation for > or = 4 days	Pre-existing neuromuscular conditions or other known myopathies	Clinical examination and electrophysiological examination	Examinations on days 4,8 and 14 after initiation of mechanical ventilation Clinical examination: functional disability score (FDS) calculated Electrophysiological examination: electromyography Diagnosis: Reduced Compound Muscle and sensory nerve action potentials with fibrillation potentials and positive sharp waves, low FDS	(16/28) 57%
19. Montero et al. [64]	PC Single centre Spain	73	Septic patients with evidence of multi-organ dysfunction and mechanical ventilation for ≥ ten days	< 18 years or > 80 years old, comorbidities such as other known myopathies, infection with HIV, renal failure, liver cirrhosis	Electrophysiological examination	Electrophysiological examinations on day 10 and day 21 from initiation of mechanical ventilation Diagnosis: reduced CMAP and SNAP amplitudes with fibrillation potentials	(50/73) 69%
20. Tepper et al. [69]	PC Single centre Netherlands	25	Diagnosis of septic shock	Age > 80 years old, pre-existing neuromuscular conditions, neuropathies/myopathies, renal disease, diabetes, alcohol abuse	Electrophysiological examination	Electrophysiological examination within 72 h of admission Diagnosis: Reduced velocity, CMAP and spontaneous activity presence	(19/25) 76%

*PC* prospective cohort, *RC* retrospective cohort, *RCT* randomised controlled trial, *ICU* intensive care unit, *MRC sum* muscle power assessment scale, *SAPS* simplified acute physiology score

The observational studies assessed with the Newcastle–Ottawa Scale were found to have relatively low risk of bias being all good quality (6*). The randomised controlled study was assessed with the ROB scale, and we found fair risk of bias specifically about blinding of participants and personnel and blinding of outcome assessment (refer to the Additional File 1).

## Outcomes

### Assessment methods

We analysed the methods used to assess muscle wasting and found that of the 33 studies that measured muscle size, 28 (85%) studies used ultrasound [11–17, 24–31, 31–44] and 5 studies (15%) used computed tomography (CT) [45–49] at different time points. Additional methods used in conjunction with ultrasound and CT were the ratio of protein to DNA and histopathological analyses [11], bioelectrical impedance analysis [31], and the urea-to-creatinine ratio in blood [48]. This reveals a high degree of inconsistency in assessing muscle mass as different studies analyse different muscles at different time points during critical illness. The main muscles assessed using ultrasound were rectus femoris, quadriceps muscle, and biceps brachii with measurements taken for cross-sectional area or thickness. The areas measured on CT were the skeletal muscle cross-sectional area at the third vertebrae (L3) level and the cross-sectional area of the femoral muscle volume using sagittal direction integration.

### Changes in muscle mass

During the first week of critical illness, patients lost on average every day −1.75% (95% CI −2.05, −1.45) of their rectus femoris thickness and −2.10% (95% CI −3.17, −1.02) of their rectus femoris cross-sectional area, respectively. Quadriceps muscle thickness decreased by −1.82% (95% CI −2.97, −0.66) each day. The daily loss in biceps brachii muscle cross-sectional area was −2.23% (95% CI −2.60, −1.80) and −1.64% (95% CI −3.09, 0.19) for biceps brachii thickness.

**Fig. 2:**
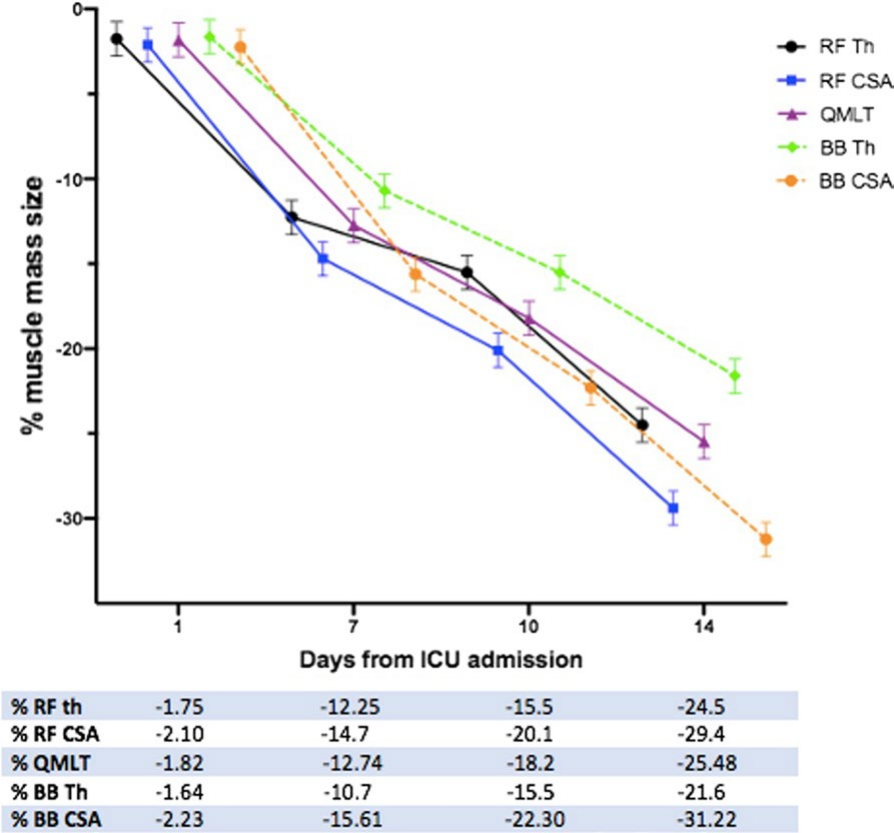
Loss in muscle mass from day 1 to day 14 of ICU admission. Abbreviations: percentage, %; rectus femoris: RF; cross-sectional area: CSA, thickness: Th, quadriceps muscle layer thickness: QMLT; biceps brachii: BB

Four studies measured [16, 25, 37, 40] both rectus femoris cross-sectional area and thickness and highlighted that thickness measurement can significantly underestimate muscle loss compared with cross-sectional area (*p* < 0.001). This was also similar for bicep brachii [37]. The loss in muscle mass for all the muscles measured over the course of ICU stay is presented in Fig. 2.

Four studies assessed skeletal muscle mass cross-sectional area at lumbar 3 level on CT scans differently. One study [45] found a reduction of −21.9 (−29.9 to −13.9) cm^2^ [(149.9 ± 38.8 cm^2^ versus 127.9 ± 38.4 cm^2^), *p* < 0.001] equal to 15% loss in muscle mass during the first week in ICU. The second study [47] reported a change of −2.09 (± 6.96) cm^2^/m^2^ (CT 1 48.73 ± 12.57 cm^2^/m^2^ versus CT 2 46.64 ± 10.64 cm^2^/m^2^; *p* = 0.183), equal to 4.29% loss over 7 to 14 days of admission. The third study [49] noted a skeletal muscle cross-sectional area reduction of 5.85% at 25 days equal to a difference of −1.00 (−1.32, 3.32) cm^2^/m^2^ (baseline: 17.1 ± 5.4 vs. day 25: 16.1 ± 5.2) from baseline. The fourth study compared an initial CT on admission versus a repeated CT taken within 1–9 days or after 10 days of ICU stay and measured the urea/creatinine ratio [48].

The L4 psoas and L3 muscle cross-sectional area both progressively decrease over time (*R*^2^ 0.64 and 0.59, respectively), and the skeletal muscle wasting is accompanied by elevated urea/creatinine ratio.

One RCT [46] assessing femoral muscle volume in patients receiving high-protein versus medium-protein intake found that muscle volume loss at day 10 (assessed using CT scan) was significantly lower in patients receiving high-protein (high-protein group: 12.9 ± 8.5% versus medium-protein group: 16.9 ± 7.0%, *p* = 0.0059). Total energy delivery was around 20 kcal/kg/day in both groups, but protein delivery was 1.5 g/kg/day and 0.8 g/kg/day. Early active rehabilitation was also provided to both groups. A second RCT [44] comparing the effect of continuous versus intermittent feeding found that muscle loss at day 10 (i.e. rectus femoris muscle cross-sectional area determined by ultrasound) was similar between arms (−1.1% [95% CI, −6.1% to −4.0%]; *p* = 0.676). Intermittently fed patients received 80% or more of target protein (OR, 1.52 [1.16–1.99]; *p* < 0.001) and energy (OR, 1.59 [1.21–2.08]; *p* = 0.001).

### Prevalence of ICU-acquired weakness

Twenty studies analysed the prevalence of ICU-acquired muscle weakness [3, 25, 50–61, 63–69], of these 9 used the MRC sum score, which is a validated clinical examination for assessment of muscle strength and power of upper and lower extremities, 6 used electrophysiological examination, and 5 used both. The overall prevalence of ICU-AW in the twenty selected studies is 48% (95% CI 39%, 56%). This varied across studies, from 43% (95% CI 31%, 55%) in those using the MRC sum score clinical examination alone to 55% (95% CI 41%, 69%) in studies using solely electrophysiological examination. Studies using MRC sum score clinical examination combined with electrophysiological examination had a prevalence of ICU-AW equal to 48% (95% CI 31%, 65%).

### Outcomes associated with muscle wasting

A meta-analysis of outcomes associated with muscle wasting was not possible as studies assessed various outcomes differently. For example, the outcome of mortality was not equally assessed and in a study was evaluated 60-day mortality [70], or in-hospital mortality [28], or mortality in ICU [71].

A study noted that patients with multi-organ failure lost muscle mass early and that the loss was more severe when  compared to patients with single organ failure [11]. In patients with sepsis and septic shock, the changes of rectus femoris cross-sectional area were reported to be significantly higher (17.5%) in mechanically ventilated patients compared to those without ventilation (10.76%), *p* = 0.001 [27]. Early decline in biceps brachii mass was found a predictor for mortality [28]. Additionally, a study noted that over the first week of critical illness, every 1% loss of quadriceps femoris muscle thickness was associated with 5% increase in 60-day mortality [adjusted OR 0.95 (95% CI 0.90, 0.99) *p* = 0.023] [15]. A logistic regression analysis noted that patients who lost more than 10% of quadriceps femoris muscle thickness at day 7 had higher probability to remain on mechanical ventilation [HR: 2.1 (95% CI, 1.1, 3.8); *p* = 0.017] [14]. During the first week in intensive care, more than 10% loss of rectus femoris cross-sectional area was associated with longer ICU length of stay (*p* = 0.038), hospital length of stay (*p* = 0.014), and mechanical ventilation time (*p* = 0.05) [12].

In patients with sepsis and acute respiratory distress syndrome, muscle wasting during the first 7 days of ICU was found to be a predictor for ICU-acquired weakness (area under the curve = 0.912) [16]. Patients presenting with muscle wasting and ICU-acquired weakness on day 3 had longer mechanical ventilation time (*p* = 0.006) and ECMO (*p* = 0.025) compared to those with no ICU-AW [13].

## Discussion

In this systematic review, we pooled results from 53 studies from international settings including 3251 critically ill patients. Our main findings are that (1) 85% of studies used ultrasound to assess muscle mass with measurements taken at the rectus femoris, quadriceps muscle and biceps brachii cross-sectional area or thickness; (2) during the first week of critical illness, patients lose roughly 2% of muscle mass per day, and muscle mass decreases over the course of the ICU stay; and (3) half of the critically ill patients have ICU-acquired weakness.

There is no consensus on how to quantify muscular changes in critically ill patients. In our analysis, ultrasound was the most frequently used method. This is probably because ultrasound devices are portable and can be used directly at the bedside of the patient. In contrast, CT requires transferring the patient to the scanner, which is risky and may not always be possible depending on the clinical stability of the critically ill patient.

The use of ultrasound is reliable (intraclass correlation coefficient, > 0.75 for all comparisons) when considering interobserver correlation for quantitative analysis of muscle parameters in critically ill patients [72]. Use and interpretation of ultrasound measurements are not without challenges, as there is considerable methodological variability in the measurement technique to quantify muscle mass. Specifically, the cross-sectional area and muscle layer thickness are different measurements and do not account for the same volume. It has been shown that for assessment of rectus femoris measuring the muscle layer thickness significantly underestimated ICU muscle wasting compared with cross-sectional area [40]. Furthermore, ultrasound-based quadriceps muscle layer thickness (QMLT) did not accurately estimate muscle loss when compared to quantifications of computed tomography (CT)-based muscle cross-sectional area (CSA) [73]. A study found that measuring cross-sectional area may be a more reliable proxy for muscle strength and could be used as a biomarker for proximal lower-limb muscle loss and knee extensor weakness during early critical illness in settings where volitional and non-volitional muscle strength measurements are challenging [40].

The incidence rate of ICU-AW was high (48%), and our findings may be an under-representation of the actual prevalence, as this depends upon the diagnostic evaluation used. We noted that electrophysiological examination resulted in the detection of more individuals with ICU-AW. This is potentially attributed to the fact that clinical examinations have a certain extent of subjectivity, and the diagnosis is partially determined by the clinician’s decision. On the other hand, electrophysiological assessments are standardised with clear cut-off values and instructions for the diagnosis. However, this difference can also result from methodological dissimilarity in assessing ICU-AW, such as the timing of diagnosis, the lack of homogeneity between patient populations and variable assessment frequency.

### Strength and limitations

Our systematic review is the first to quantify the overall rate of muscle loss in critically ill, but has limitations. Firstly, there was a high degree of inconsistency in assessing muscle mass since studies assessed different muscles and different measurements methods at different time points during critical illness. Therefore, a differentiation of muscle loss for individual illnesses was not possible and thus remained unanswered. Second, the pre-admission baseline characteristics and patient functional state were limited. Consequently, it was not possible to assess the impact on muscle loss by severity of critical illness or pre-existing comorbidities. Finally, the studies inconsistency in assessing outcomes made a meta-analysis of outcomes associated with muscle wasting not possible. The recent CONCISE Delphi consensus should provide further guidance for authors assessing outcomes related to muscle wasting [74].

## Conclusion

Critically ill patients suffer from early and marked muscle wasting. Ultrasound is the most used assessment tool in evaluating loss in muscle mass over time. The muscle mass is about 2% per day, but this rate is different between muscles and depends upon the measurement taken. The prevalence of ICU-AW is 50% amongst critically ill and those have worst outcomes.

## Supplementary Information

**Additional file 1**. **Table 1.** Quality and risk of bias assessment using the Newcastle-Ottawa Scale (NOS) for observational studies assessing muscle wasting. **Table 2.** Quality and risk of bias assessment using the Newcastle-Ottawa Scale (NOS) for observational studies assessing ICU-acquired weakness. **Figure 1.** Risk of Bias.

## Data Availability

Supplementary materials are available and can be accessed online.

## References

[1] Fan E, Cheek F, Chlan L, Gosselink R, Hart N, Herridge MS, Hopkins RO, Hough CL, Kress JP, Latronico N, Moss M, Needham DM, Rich MM, Stevens RD, Wilson KC, Winkelman C, Zochodne DW, Ali NA; ATS Committee on ICU-acquired Weakness in Adults; American Thoracic Society. An official American Thoracic Society Clinical Practice guideline: the diagnosis of intensive care unit-acquired weakness in adults. Am J Respir Crit Care Med. 2014;190(12):1437–46. doi:10.1164/rccm.201411-2011ST. 10.1164/rccm.201411-2011ST 25496103

[2] Schefold JC, Bierbrauer J, Weber-Carstens S (2010). Intensive care unit-acquired weakness (ICUAW) and muscle wasting in critically ill patients with severe sepsis and septic shock. J Cachexia Sarcopenia Muscle.

[3] de Letter MA, Schmitz PI, Visser LH, Verheul FA, Schellens RL, Op de Coul DA, et al. Risk factors for the development of polyneuropathy and myopathy in critically ill patients. Crit Care Med. 2001;29(12):2281–6. 10.1097/00003246-200112000-00008 11801825

[4] Eikermann M, Koch G, Gerwig M, Ochterbeck C, Beiderlinden M, Koeppen S, Neuhäuser M, Peters J (2006). Muscle force and fatigue in patients with sepsis and multiorgan failure. Intensive Care Med.

[5] Weber-Carstens S, Deja M, Koch S, Spranger J, Bubser F, Wernecke KD, Spies CD, Spuler S, Keh D (2010). Risk factors in critical illness myopathy during the early course of critical illness: a prospective observational study. Crit Care.

[6] Kress JP, Hall JB (2014). ICU-acquired weakness and recovery from critical illness. N Engl J Med.

[7] Friedrich O, Reid MB, Van den Berghe G, Vanhorebeek I, Hermans G, Rich MM, Larsson L (2015). The sick and the weak: neuropathies/myopathies in the critically Ill. Physiol Rev.

[8] Crossland H, Skirrow S, Puthucheary ZA, Constantin-Teodosiu D, Greenhaff PL (2019). The impact of immobilisation and inflammation on the regulation of muscle mass and insulin resistance: different routes to similar end-points. J Physiol.

[9] Puthucheary ZA, Astin R, Mcphail MJW, Saeed S, Pasha Y, Bear DE, Constantin D, Velloso C, Manning S, Calvert L, Singer M, Batterham RL, Gomez-Romero M, Holmes E, Steiner MC, Atherton PJ, Greenhaff P, Edwards LM, Smith K, Harridge SD, Hart N, Montgomery HE (2018). Metabolic phenotype of skeletal muscle in early critical illness. Thorax.

[10] Latronico N, Bolton CF (2011). Critical illness polyneuropathy and myopathy: a major cause of muscle weakness and paralysis. Lancet Neurol.

[11] Puthucheary ZA, Rawal J, McPhail M, Connolly B, Ratnayake G, Chan P (2013). Acute skeletal muscle wasting in critical illness. JAMA.

[12] Kemp PR, Paul R, Hinken AC, Neil D, Russell A, Griffiths MJ (2020). Metabolic profiling shows pre-existing mitochondrial dysfunction contributes to muscle loss in a model of ICU-acquired weakness. J Cachexia Sarcopenia Muscle.

[13] Dimopoulos S, Raidou V, Elaiopoulos D, Chatzivasiloglou F, Markantonaki D, Lyberopoulou E (2020). Sonographic muscle mass assessment in patients after cardiac surgery. World J Cardiol.

[14] Toledo DO, Freitas BJ, Dib R, Pfeilsticker F, Santos DMD, Gomes BC (2021). Peripheral muscular ultrasound as outcome assessment tool in critically ill patients on mechanical ventilation: an observational cohort study. Clin Nutr ESPEN.

[15] Lee ZY, Ong SP, Ng CC, Yap CSL, Engkasan JP, Barakatun-Nisak MY (2021). Association between ultrasound quadriceps muscle status with premorbid functional status and 60-day mortality in mechanically ventilated critically ill patient: a single-center prospective observational study. Clin Nutr.

[16] Mayer KP, Thompson Bastin ML, Montgomery-Yates AA, Pastva AM, Dupont-Versteegden EE, Parry SM (2020). Acute skeletal muscle wasting and dysfunction predict physical disability at hospital discharge in patients with critical illness. Crit Care.

[17] Palakshappa JA, Reilly JP, Schweickert WD, Anderson BJ, Khoury V, Shashaty MG (2018). Quantitative peripheral muscle ultrasound in sepsis: muscle area superior to thickness. J Crit Care.

[18] Aromataris E, Munn Z (Editors). JBI manual for evidence synthesis. JBI, 2020. Available from https://synthesismanual.jbi.global. 10.46658/JBIMES-20-01.

[19] Page MJ, McKenzie JE, Bossuyt PM (2021). The PRISMA 2020 statement: an updated guideline for reporting systematic reviews. Syst Rev.

[20] Liberati A, Altman DG, Tetzlaff J (2009). The PRISMA statement for reporting systematic reviews and meta-analyses of studies that evaluate healthcare interventions: explanation and elaboration. BMJ.

[21] Seymour JM, Ward K, Sidhu PS (2009). Ultrasound measurement of rectus femoris cross-sectional area and the relationship with quadriceps strength in COPD. Thorax.

[22] Dechartres A, Trinquart L, Boutron I, Ravaud P (2013). Influence of trial sample size on treatment effect estimates: meta-epidemiological study. BMJ.

[23] Higgins JPT, Thomas J, Chandler J, Cumpston M, Li T, Page MJ, Welch VA (editors). Cochrane Handbook for Systematic Reviews of Interventions version 6.3 (updated February 2022). Cochrane, 2022. Available from www.training.cochrane.org/handbook.

[24] Pardo E, El Behi H, Boizeau P, Verdonk F, Alberti C, Lescot T (2018). Reliability of ultrasound measurements of quadriceps muscle thickness in critically ill patients. BMC Anesthesiol.

[25] Parry SM, El-Ansary D, Cartwright MS, Sarwal A, Berney S, Koopman R (2015). Ultrasonography in the intensive care setting can be used to detect changes in the quality and quantity of muscle and is related to muscle strength and function. J Crit Care.

[26] Zhang W, Wu J, Gu Q, Gu Y, Zhao Y, Ge X (2021). Changes in muscle ultrasound for the diagnosis of intensive care unit acquired weakness in critically ill patients. Sci Rep.

[27] Borges RC, Barbeiro HV, Barbeiro DF, Soriano FG (2020). Muscle degradation, vitamin D and systemic inflammation in hospitalized septic patients. J Crit Care.

[28] Nakanishi N, Oto J, Tsutsumi R, Akimoto Y, Nakano Y, Nishimura M (2020). Upper limb muscle atrophy associated with in-hospital mortality and physical function impairments in mechanically ventilated critically ill adults: a two-center prospective observational study. J Intensive Care.

[29] Nakanishi N, Tsutsumi R, Hara K, Takashima T, Nakataki E, Itagaki T (2020). Urinary titin is a novel biomarker for muscle atrophy in nonsurgical critically Ill patients: a two-center, prospective observational study. Crit Care Med.

[30] Borges RC, Soriano FG (2019). Association between muscle wasting and muscle strength in patients who developed severe sepsis and septic shock. Shock.

[31] Nakanishi N, Tsutsumi R, Okayama Y, Takashima T, Ueno Y, Itagaki T (2019). Monitoring of muscle mass in critically ill patients: comparison of ultrasound and two bioelectrical impedance analysis devices. J Intensive Care.

[32] Trung TN, Duoc NVT, Nhat LTH, Yen LM, Hao NV, Truong NT (2019). Functional outcome and muscle wasting in adults with tetanus. Trans R Soc Trop Med Hyg.

[33] Wandrag L, Brett SJ, Frost GS, Bountziouka V, Hickson M (2019). Exploration of muscle loss and metabolic state during prolonged critical illness: Implications for intervention?. PLoS ONE.

[34] Hadda V, Kumar R, Khilnani GC, Kalaivani M, Madan K, Tiwari P (2018). Trends of loss of peripheral muscle thickness on ultrasonography and its relationship with outcomes among patients with sepsis. J Intensive Care.

[35] Hayes K, Holland AE, Pellegrino VA, Mathur S, Hodgson CL (2018). Acute skeletal muscle wasting and relation to physical function in patients requiring extracorporeal membrane oxygenation (ECMO). J Crit Care.

[36] Katari Y, Srinivasan R, Arvind P, Hiremathada S (2018). Point-of-care ultrasound to evaluate thickness of rectus femoris, vastus intermedius muscle, and fat as an indicator of muscle and fat wasting in critically Ill patients in a multidisciplinary intensive care unit. Indian J Crit Care Med.

[37] Nakanishi N, Oto J, Tsutsumi R, Iuchi M, Onodera M, Nishimura M (2018). Upper and lower limb muscle atrophy in critically ill patients: an observational ultrasonography study. Intensive Care Med.

[38] Silva PE, Maldaner V, Vieira L, de Carvalho KL, Gomes H, Melo P (2018). Neuromuscular electrophysiological disorders and muscle atrophy in mechanically-ventilated traumatic brain injury patients: new insights from a prospective observational study. J Crit Care.

[39] Annetta MG, Pittiruti M, Silvestri D, Grieco DL, Maccaglia A, La Torre MF (2017). Ultrasound assessment of rectus femoris and anterior tibialis muscles in young trauma patients. Ann Intensive Care.

[40] Puthucheary ZA, McNelly AS, Jai R, Connolly B, Sidhu PS, Rowlerson A (2017). Rectus femoris cross-sectional area and muscle layer thickness: comparative markers of muscle wasting and weakness. Am J Respir Crit Care Med.

[41] Segaran E, Wandrag L, Stotz M, Terblanche M, Hickson M (2017). Does body mass index impact on muscle wasting and recovery following critical illness? A pilot feasibility observational study. J Hum Nutr Diet.

[42] Turton P, Hay R, Taylor J, Mcphee J, Welters I (2016). Human limb skeletal muscle wasting and architectural remodeling during five to ten days intubation and ventilation in critical care e an observational study using ultrasound. BMC Anesth.

[43] Reid CL, Campbell IT, Little RA (2004). Muscle wasting and energy balance in critical illness. Clin Nutr.

[44] McNelly AS, Bear DE, Connolly BA, Arbane G, Allum L, Tarbhai A, Cooper JA, Hopkins PA, Wise MP, Brealey D, Rooney K, Cupitt J, Carr B, Koelfat K, Damink SO, Atherton PJ, Hart N, Montgomery HE, Puthucheary ZA (2020). Effect of intermittent or continuous feed on muscle wasting in critical illness: a phase 2 clinical trial. Chest.

[45] Lambell KJ, Tierney AC, Wang JC, Nanjayya V, Forsyth A, Goh GS (2021). Comparison of ultrasound-derived muscle thickness with computed tomography muscle cross-sectional area on admission to the intensive care unit: a pilot cross-sectional study. JPEN J Parenter Enteral Nutr.

[46] Nakamura K, Nakano H, Naraba H, Mochizuki M, Takahashi Y, Sonoo T, Hashimoto H, Morimura N (2021). High protein versus medium protein delivery under equal total energy delivery in critical care: a randomized controlled trial. Clin Nutr.

[47] Dusseaux MM, Antoun S, Grigioni S, Béduneau G, Carpentier D, Girault C (2019). Skeletal muscle mass and adipose tissue alteration in critically ill patients. PLoS ONE.

[48] Haines RW, Zolfaghari P, Wan Y, Pearse RM, Puthucheary Z, Prowle JR (2019). Elevated urea-to-creatinine ratio provides a biochemical signature of muscle catabolism and persistent critical illness after major trauma. Intensive Care Med.

[49] Jung B, Nougaret S, Conseil M, Coisel Y, Futier E, Chanques G (2014). Sepsis is associated with a preferential diaphragmatic atrophy: a critically ill patient study using tridimensional computed tomography. Anesthesiology.

[50] Van Aerde N, Meersseman P, Debaveye Y, Wilmer A, Gunst J, Casaer MP, Bruyninckx F, Wouters PJ, Gosselink R, Van den Berghe G, Hermans G (2020). Five-year impact of ICU-acquired neuromuscular complications: a prospective, observational study. Intensive Care Med.

[51] Diaz Ballve LP, Dargains N, Urrutia Inchaustegui JG, Bratos A, Milagros Percaz M, Bueno Ardariz C, et al. Weakness acquired in the intensive care unit. Incidence, risk factors and their association with inspiratory weakness. Observational cohort study. Rev Bras Ter Intensiva. 2017;29(4):466–75. 10.5935/0103-507X.20170063 PMC5764559 29236843

[52] Nguyen The L, Nguyen HuuC (2015). Critical illness polyneuropathy and myopathy in a rural area in Vietnam. J Neurol Sci.

[53] Hough CL, Lieu BK, Caldwell ES (2011). Manual muscle strength testing of critically ill patients: feasibility and interobserver agreement. Crit Care.

[54] Brunello AG, Haenggi M, Wigger O, Porta F, Takala J, Jakob SM (2010). Usefulness of a clinical diagnosis of ICU-acquired paresis to predict outcome in patients with SIRS and acute respiratory failure. Intensive Care Med.

[55] Weber-Carstens S, Koch S, Spuler S, Spies CD, Bubser F, Wernecke KD (2009). Nonexcitable muscle membrane predicts intensive care unit-acquired paresis in mechanically ventilated, sedated patients. Crit Care Med.

[56] Ahlbeck K, Fredriksson K, Rooyackers O, Maback G, Remahl S, Ansved T (2009). Signs of critical illness polyneuropathy and myopathy can be seen early in the ICU course. Acta Anaesthesiol Scand.

[57] Sharshar T, Bastuji-Garin S, Stevens RD, Durand MC, Malissin I, Rodriguez P (2009). Presence and severity of intensive care unit-acquired paresis at time of awakening are associated with increased intensive care unit and hospital mortality. Crit Care Med.

[58] Nanas S, Kritikos K, Angelopoulos E, Siafaka A, Tsikriki S, Poriazi M (2008). Predisposing factors for critical illness polyneuromyopathy in a multidisciplinary intensive care unit. Acta Neurol Scand.

[59] Ali NA, O’Brien JM, Hoffmann SP, Phillips G, Garland A, Finley JC (2008). Acquired weakness, handgrip strength, and mortality in critically ill patients. Am J Respir Crit Care Med.

[60] Latronico N, Bertolini G, Guarneri B, Botteri M, Peli E, Andreoletti S (2007). Simplified electrophysiological evaluation of peripheral nerves in critically ill patients: the Italian multi-centre CRIMYNE study. Crit Care.

[61] Amaya-Villar R, Garnacho-Montero J, Garcia-Garmendia JL, Madrazo-Osuna J, Garnacho-Montero MC, Luque R (2005). Steroid-induced myopathy in patients intubated due to exacerbation of chronic obstructive pulmonary disease. Intensive Care Med.

[62] Bednarik J, Vondracek P, Dusek L, Moravcova E, Cundrle I. Risk factors for critical illness polyneuromyopathy. J Neurol. 2005;252(3):343–51. 10.1007/s00415-005-0654-x 15791390

[63] Bednarik J, Vondracek P, Dusek L, Moravcova E, Cundrle I (2005). Risk factors for critical illness polyneuromyopathy. J Neurol.

[64] Garnacho-Montero J, Amaya-Villar R, Garcia-Garmendia JL, Madrazo-Osuna J, Ortiz-Leyba C (2005). Effect of critical illness polyneuropathy on the withdrawal from mechanical ventilation and the length of stay in septic patients. Crit Care Med.

[65] Bercker S, Weber-Carstens S, Deja M, Grimm C, Wolf S, Behse F (2005). Critical illness polyneuropathy and myopathy in patients with acute respiratory distress syndrome. Crit Care Med.

[66] De Jonghe B, Sharshar T, Lefaucheur JP, Authier FJ, Durand-Zaleski I, Boussarsar M (2002). Paresis acquired in the intensive care unit: a prospective multicenter study. JAMA.

[67] Druschky A, Herkert M, Radespiel-Troger M, Druschky K, Hund E, Becker CM (2001). Critical illness polyneuropathy: clinical findings and cell culture assay of neurotoxicity assessed by a prospective study. Intensive Care Med.

[68] Garnacho-Montero J, Madrazo-Osuna J, Garcia-Garmendia JL, Ortiz-Leyba C, Jimenez-Jimenez FJ, Barrero-Almodovar A (2001). Critical illness polyneuropathy: risk factors and clinical consequences: a cohort study in septic patients. Intensive Care Med.

[69] Tepper M, Rakic S, Haas JA, Woittiez AJ (2000). Incidence and onset of critical illness polyneuropathy in patients with septic shock. Neth J Med.

[70] Lee ZY, Ong SP, Ng CC, Yap CSL, Engkasan JP, Barakatun-Nisak MY, Heyland DK, Hasan MS (2021). Association between ultrasound quadriceps muscle status with premorbid functional status and 60-day mortality in mechanically ventilated critically ill patient: a single-center prospective observational study. Clin Nutr.

[71] Dusseaux MM, Antoun S, Grigioni S, Béduneau G, Carpentier D, Girault C, Grange S, Tamion F (2019). Skeletal muscle mass and adipose tissue alteration in critically ill patients. PLoS ONE.

[72] Sarwal A, Parry SM, Berry MJ, Hsu FC, Lewis MT, Justus NW, Morris PE, Denehy L, Berney S, Dhar S, Cartwright MS (2015). Interobserver reliability of quantitative muscle sonographic analysis in the critically Ill population. J Ultrasound Med.

[73] Paris MT, Mourtzakis M, Day A, Leung R, Watharkar S, Kozar R, Earthman C, Kuchnia A, Dhaliwal R, Moisey L, Compher C, Martin N, Nicolo M, White T, Roosevelt H, Peterson S, Heyland DK (2017). Validation of bedside ultrasound of muscle layer thickness of the quadriceps in the critically Ill patient (VALIDUM study). JPEN J Parenter Enteral Nutr.

[74] Davies TW, van Gassel RJJ, van de Poll M, Gunst J, Casaer MP, Christopher KB, Preiser JC, Hill A, Gundogan K, Reintam-Blaser A, Rousseau AF, Hodgson C, Needham DM, Castro M, Schaller S, McClelland T, Pilkington JJ, Sevin CM, Wischmeyer PE, Lee ZY, Govil D, Li A, Chapple L, Denehy L, Montejo-González JC, Taylor B, Bear DE, Pearse R, McNelly A, Prowle J, Puthucheary ZA (2022). Core outcome measures for clinical effectiveness trials of nutritional and metabolic interventions in critical illness: an international modified Delphi consensus study evaluation (CONCISE). Crit Care.

