# Supplementary material for: The rate and assessment of muscle wasting during critical illness: a systematic review and meta-analysis
Source: Crit Care. 2023 Jan 3;27:2. doi: 10.1186/s13054-022-04253-0 (PMC9808763; doi:10.1186/s13054-022-04253-0)
Supplement: Supplementary file 1 — Additional file 1. Table 1. Quality and risk of bias assessment using the Newcastle-Ottawa Scale (NOS) for observational studies assessing muscle wasting. Table 2. Quality and risk of bias assessment using the Newcastle-Ottawa Scale (NOS) for observational studies assessing ICU-acquired weakness. Figure 1. Risk of Bias. [file 13054_2022_4253_MOESM1_ESM.docx]

**Supplementary materials**

Table 1. Quality and risk of bias assessment using the Newcastle-Ottawa Scale (NOS) for observational studies assessing muscle wasting.

| **Study ID** | **Selection** | | | | **Comparability** | **Outcome** | | | **Total**  **(9*)** |
| --- | --- | --- | --- | --- | --- | --- | --- | --- | --- |
|  | Representativeness of the exposed cohort (*) | Selection of non-exposed cohort (*) | Ascertainment of exposure (*) | Demonstration that outcome of interest was not present at start of study (*) | Comparability of cohorts (**) | Assessment of outcome (*) | Follow up long enough for outcomes to occur (*) | Adequacy of follow up (*) |  |
| Lambell, 2021 | * |  | * | * |  | * | * | * | 6 |
| Lee, 2021 | * |  | * | * |  | * | * | * | 6 |
| Toledo, 2021 | * |  | * | * |  | * | * | * | 6 |
| Zhang, 2021 | * |  | * | * |  | * | * | * | 6 |
| Borges, 2020 | * |  | * | * |  | * | * | * | 6 |
| Dimopoulos, 2020 | * |  | * | * |  | * | * | * | 6 |
| Kemp, 2020 | * |  | * | * |  | * | * | * | 6 |
| Mayer, 2020 | * |  | * | * |  | * | * | * | 6 |
| Nakanishi, 2020 (a) | * |  | * | * |  | * | * | * | 6 |
| Nakanishi, 2020 (b) | * |  | * | * |  | * | * | * | 6 |
| Borges, 2019 | * |  | * | * |  | * | * | * | 6 |
| Dusseaux, 2019 | * |  | * | * |  | * | * | * | 6 |
| Haines, 2019 | * |  | * | * |  | * | * | * | 6 |
| Nakanishi, (c) 2019 | * |  | * | * |  | * | * | * | 6 |
| Trung, 2019 | * |  | * | * |  | * | * | * | 6 |
| Vivier, 2019 | * |  | * | * |  | * | * |  | 6 |
| Wandrag, 2019 | * |  | * | * |  | * | * | * | 6 |
| Hadda, 2018 | * |  | * | * |  | * | * | * | 6 |
| Hayes, 2018 | * |  | * | * |  | * | * | * | 6 |
| Katari, 2018 | * |  | * | * |  | * | * | * | 6 |
| Nakanishi, (d) 2018 | * |  | * | * |  | * | * | * | 6 |
| Palakshappa,  2018 | * |  |  | * |  | * | * | * | 6 |
| Pardo, 2018 | * |  | * | * |  | * | * | * | 6 |
| Silva, 2018 | * |  | * | * |  | * | * | * | 6 |
| Annetta, 2017 | * |  | * | * | * | * | * | * | 7 |
| Puthucheary,  2017 | * |  | * | * | * | * | * | * | 7 |
| Segaran, 2016 | * |  | * | * | * | * | * | * | 7 |
| Turton,  2016 | * |  | * | * | * | * | * | * | 7 |
| Parry, 2015 | * |  | * | * | * | * | * | * | 7 |
| Jung, 2014 | * |  | * | * | * | * | * | * | 7 |
| Puthucheary, 2013 | * |  | * | * | * | * | * | * | 7 |
| Reid, 2004 | * |  | * | * | * | * | * | * | 7 |

Table 2. Quality and risk of bias assessment using the Newcastle-Ottawa Scale (NOS) for observational studies assessing ICU-acquired weakness.

| **Study ID** | **Selection** | | | | **Comparability** | **Outcome** | | | **Total**  **(9*)** |
| --- | --- | --- | --- | --- | --- | --- | --- | --- | --- |
|  | Representativeness of the exposed cohort (*) | Selection of non-exposed cohort (*) | Ascertainment of exposure (*) | Demonstration that outcome of interest was not present at start of study (*) | Comparability of cohorts (**) | Assessment of outcome (*) | Follow up long enough for outcomes to occur (*) | Adequacy of follow up (*) |  |
| 1. Van Aerde et al. 2020 | * |  | * | * |  | * | * | * | 6 |
| 2. Ballve et al. 2017 | * |  | * | * |  | * | * | * | 6 |
| 3. Nguyen et al. 2015 | * |  | * | * |  | * | * | * | 6 |
| 4. Parry et al. 2015 | * |  | * | * |  | * | * | * | 6 |
| 5. Hough et al. 2011 | * |  | * | * |  | * | * | * | 6 |
| 6. Brunello et al. 2010 | * |  | * | * |  | * | * | * | 6 |
| 7. Carstens et al. 2009 | * |  | * | * |  | * | * | * | 6 |
| 8. Sharshar et al. 2009 | * |  | * | * |  | * | * | * | 6 |
| 9. Nanas et al. 2008 | * |  | * | * |  | * | * | * | 6 |
| 10. Ali et al.  2008 | * |  | * | * |  | * | * | * | 6 |
| 11. Latronico et al. 2007 | * |  | * | * |  | * | * | * | 6 |
| 12. Villar et al. 2005 | * |  | * | * |  | * | * | * | 6 |
| 13. Bedranik et al.  2005 | * |  | * | * |  | * | * | * | 6 |
| 14. Montero et al.  2005 | * |  | * | * |  | * | * | * | 6 |
| 15. Bercker et al.  2005 | * |  | * | * |  | * | * | * | 6 |
| 16. Jonghe et al.  2002 | * |  | * | * |  | * | * | * | 6 |
| 17. Letter et al.  2001 | * |  | * | * |  | * | * | * | 6 |
| 18. Druschky et al.  2001 | * |  | * | * |  | * | * | * | 6 |
| 19. Montero et al.  2001 | * |  | * | * |  | * | * | * | 6 |
| 20. Tepper et al.  2000 | * |  | * | * |  | * | * | * | 6 |


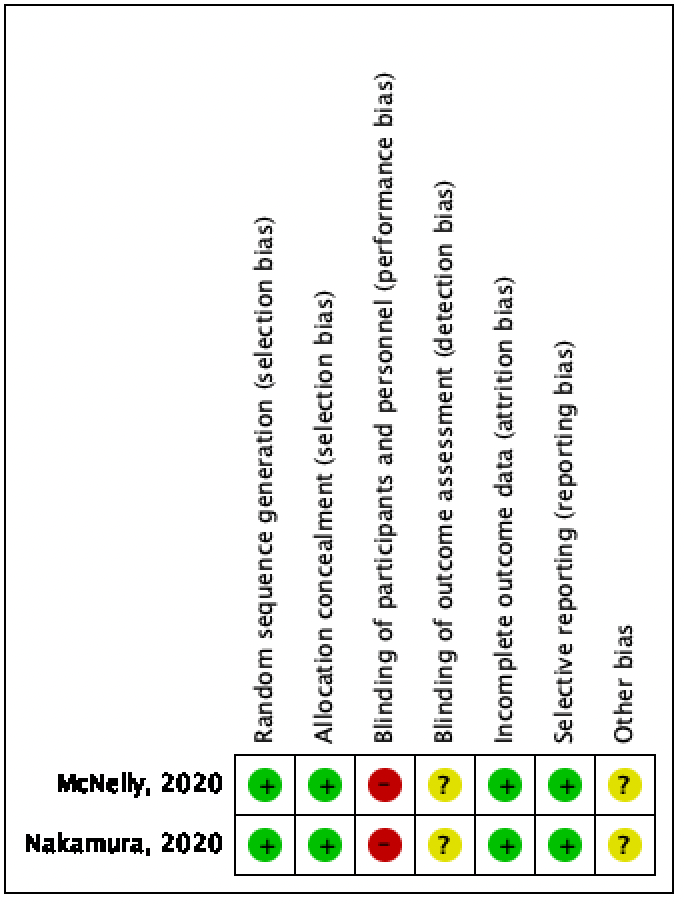


Figure 1. Risk of Bias
